# Self-rated health among undocumented and newly regularized migrants in Geneva: a cross-sectional study

**DOI:** 10.1186/s12889-021-11239-0

**Published:** 2021-06-23

**Authors:** Julien Fakhoury, Claudine Burton-Jeangros, Idris Guessous, Liala Consoli, Aline Duvoisin, Yves Jackson

**Affiliations:** 1 Swiss NCCR Lives, University of Geneva, Geneva, Switzerland; 2 Centre interfacultaire de gerontologie et d’etudes des vulnerabilites (CIGEV), University of Geneva, Geneva, Switzerland; 3 Institute of sociological research, University of Geneva, Geneva, Switzerland; 4 Division of Primary Care Medicine, Geneva University Hospital and University of Geneva, Geneva, Switzerland

**Keywords:** Undocumented migrants, Self-rated health, Legal status regularization, Geneva, Switzerland

## Abstract

**Background:**

In Europe, knowledge about the social determinants of health among undocumented migrants is scarce. The canton of Geneva, Switzerland, implemented in 2017–2018 a pilot public policy aiming at regularizing undocumented migrants. We sought to test for associations between self-rated health, proven eligibility for residence status regularization and social and economic integration.

**Methods:**

This paper reports data from the first wave of the Parchemins Study, a prospective study whose aim is to investigate the effect of residence status regularization on undocumented migrants’ living conditions and health. The convenience sample included undocumented migrants living in Geneva for at least 3 years. We categorized them into those who were in the process of receiving or had just been granted a residence permit (eligible or newly regularized) and those who had not applied or were ineligible for regularization (undocumented). We conducted multivariate regression analyses to determine factors associated with better self-rated health, i.e., with excellent/very good vs. good/fair/poor self-rated health. Among these factors, measures of integration, social support and economic resources were included.

**Results:**

Of the 437 participants, 202 (46%) belonged to the eligible or newly regularized group. This group reported better health more frequently than the undocumented group (44.6% versus 28.9%, *p*-value < .001), but the association was no longer significant after adjustment for social support and economic factors (odds ratio (OR): 1.12; 95% confidence interval (CI): 0.67–1.87). Overall, better health was associated with larger social networks (OR: 1.66; 95% CI: 1.04–2.64). This association remained significant even after adjusting for health-related variables.

**Conclusion:**

At the onset of the regularization program, access to regularization was not associated with better self-rated health. Policies aiming at favouring undocumented migrants’ inclusion and engagement in social networks may promote better health. Future research should investigate long-term effects of residence status regularization on self-rated health.

**Supplementary Information:**

The online version contains supplementary material available at 10.1186/s12889-021-11239-0.

## Background

In recent decades, non-regulated migratory flows have become a global phenomenon [1]. The latest estimates suggest that 20 to 30 million migrants live in a foreign country without legal residence permit (undocumented), among whom 1.9 to 3.8 million are in Europe [1, 2]. Previous studies showed that undocumented migrants tend to present frequent health problems in the context of adverse living and working conditions [3–6]. Specifically, undocumented migrants were shown to be at high risk of occupational hazards and chronic physical illnesses [7–9]. Due to precarious legal and economic conditions, many are forced to perform degrading, physically demanding and low-paid jobs in under-regulated sectors of the labour economy [7, 10–12]. Furthermore, undocumented migrants tend to be exposed to cumulative sources of stress that affect their mental health [13, 14]. They have limited control over the social and economic factors that shape their living conditions and over the resources necessary to fulfill their personal aspirations [14–16]. Fear of deportation, social isolation, legal and economic constraints as well as language barriers frequently hinder their access to healthcare and welfare services [4, 16–18].

Mainly defined by their lack of valid residence permit in contrast to legal migrants, undocumented migrants still encompass various profiles of immigrants. Yet, their specificities in terms of available resources or integration in their host society are often overlooked. Thus, little is known about factors that enhance undocumented migrants’ resilience towards specific health risks entailed by the lack of residence permit. One hypothesis is that stabilization of social and economic conditions while being undocumented could be associated with better health. Indeed, camouflage techniques adopted to counter the risk of being deported suggest that integration with the intention of claiming regularization later in life would lower stress [19, 20]. Previous research showed that financial stability reduced the odds of reporting poor health among undocumented migrants and that differences in self-rated health (SRH) between documented and irregular migrants were partly explained by the latter social and economic disadvantages [14, 21, 22]. In the context of the Deferred Action for Childhood Arrivals Act (DACA), a public policy aiming at granting temporary residence permits to young undocumented migrants, it was found that the regularization of the residence status, though it did not affect SRH [23], had a positive impact on self-reported mental distress, mainly through the alleviation of the chronic stress related to uncertainty about the near future and better opportunities for education and personal development [23–25]. However, the generalizability of these findings to the European context remains poorly known due to the limited number of studies and their frequent limited sample size. A better understanding of factors promoting good health is crucial in designing health policies and interventions targeting this population [26].

In 2017–18, a pilot regularization policy in the Canton of Geneva (about 500′000 inhabitants), Switzerland, offered a unique opportunity to shed light on the relationships between undocumented migrants’ socioeconomic conditions, regularization of the residence status and health in a European context. This Canton hosts 10′000–15′000 undocumented migrants of whom a large proportion are economic migrants without valid residence authorization (undocumented economic migrants) [27, 28]. Failed asylum seekers only represent a small share of these undocumented migrants [28]. In that context, the local government implemented the “Operation Papyrus”, a selective regularization policy aiming at granting residence permits to undocumented economic migrants fulfilling strict criteria [29]. With the support of local associations and trade unions, these migrants could apply for a renewable residence permit if they: (1) had never been asylum seekers, (2) had been continuously living in Geneva for the last 10 years (5 years for a family with a school-aged child), (3) were working, (4) were financially independent, (5) had no criminal record and (6) were able to speak basic French (level A2) [30]. It was expected that between 1500 and 3000 undocumented economic migrants would thus be granted a residence permit.

The objective of this study is to describe, at the onset of the “Operation Papyrus”, the SRH status of undocumented economic migrants in Geneva. We were particularly interested in exploring whether undocumented economic migrants who were fully eligible for regularization reported better health than those who were not, taking into account differences in their level of integration and in their social and economic conditions.

## Methods

### Setting

This paper reports the data collected over the first wave of the Parchemins Study, a prospective study whose aim is to assess over four years the impact of the residence status regularization on the living and health conditions of undocumented economic migrants in Geneva [26].

### Participants

Recruitment of participants took place in Geneva between October 2017 and December 2018. As undocumented migrants are a hard-to-reach population, recruitment strategies were defined in order to draw a convenience sample as various as possible. They included face-to-face recruitment as well as snowball sampling [26]. The participants were mainly recruited in the community with the support of the associations and trade unions that acted as gatekeepers into the “Operation Papyrus”. The Geneva University Hospital, which provides primary care to undocumented migrants in a dedicated unit [26], served as a second recruitment site. All participants gave their informed consent in written form before participating.

The study population included undocumented economic migrants who were at least 18 years old, were not nationals of a Member State of the European Union or the European Free-Trade Association and were not current or former asylum seekers.

In order to be eligible for the study, undocumented economic migrants had to be living in Geneva without a valid residence permit for at least 3 years. Undocumented economic migrants who had already submitted a regularization application to the authorities in the context of the “Operation Papyrus” and were waiting for a legal decision were included. Migrants who had obtained a residence permit through the “Operation Papyrus” in the 3 months prior to participation were also eligible for the study, since this period was considered too short to allow significant changes in the living conditions of the recipients.

### Data collection

Data were collected through face-to-face interviews. A specific questionnaire including validated measures as well as original items was designed (Additional file 1). It was administered by trained investigators in either French, Spanish, Portuguese or English depending on participants’ preferences. Participants were asked about their (1) sociodemographic characteristics, (2) migration trajectories, (3) health, (4) economic and financial situation and (5) social relationships and participation in social activities. Responses were recorded in a mobile tablet by each participant with the investigator’s help and immediately transferred on a secured server.

Interviews took place either at the University of Geneva or at locations chosen by the participants. The study protocol was approved by the Ethics Committee of Geneva Canton, Switzerland (CCER 2017–00897).

### Measures

#### Dependent variable

The dependent variable was the single-item SRH, a widely used measure of health that has shown to be consistently associated with physical and mental morbidity and to predict mortality [31–33]. More specifically, we assessed SRH using the first item of the Short Form Survey (SF12v2), a health-related questionnaire validated in various languages [34]. Participants rated their health on a 5-point scale by answering the question “Overall, do you think your health is (1) excellent, (2) very good, (3) good, (4) fair or (5) poor?” We then dichotomized this variable to emphasize positive options, as this dichotomization has recently been found to better reflect one’s health status than coding schemes stressing negative ratings [35]. The modalities (3) good, (4) fair and (5) poor were hence used as reference and were attributed the value 0. The options (1) excellent and (2) very good were coded as 1.

The third modality (3) good was grouped with the options (4) fair and (5) poor, since we postulated that choosing (3) good over (1) excellent or (2) very good health revealed some reserves over the current state of health. Furthermore, it has been suggested that the choice between the modalities (3) good and (4) fair is particularly subject to heterogeneous reporting behaviour, a bias that leads to over/under-reporting of good compared to fair SRH [36].

#### Independent variables

We created a dichotomous variable measuring eligibility for regularization. The newly regularized migrants and the migrants whose regularization application was already submitted were put together into a “regularized or eligible for regularization group”. Undocumented economic migrants who had not submitted a regularization application or who were ineligible for the “Operation Papyrus” represented the “undocumented (or control) group”.

These regroupings were based on local practices. Undocumented economic migrants who met all six criteria of the “Operation Papyrus” at the time of their application were guaranteed they would obtain a residence permit. When they did not fulfill all the criteria, undocumented economic migrants were strongly urged not to apply to the “Operation Papyrus”, because of the high risks of being denied legalization and, as a result, being deported. We therefore assumed that all migrants who had submitted an application were fully eligible for regularization and fulfilled the regularization criteria. We also postulated that SRH would be associated with the eligibility for regularization, as attested by the application, rather than with the authorities’ decision on their application. Finally, we hypothesized that the time elapsed between the application and the decision would not impact on applicants’ SRH.

Sex, origin, age, level of education, experience of racial discrimination and recruitment site (in the community vs. in healthcare setting) were used as predictors, since they might influence SRH [37–41].

In the context of the “Operation Papyrus”, being eligible for regularization depended on the fulfillment of criteria related to the social integration of the applicants, their economic resources and their working situation. Therefore, assessing associations between the eligibility for regularization and SRH also required including variables related to social integration, economic resources and work.

Measures of social integration included the length of stay in Geneva and the self-rated level of oral French proficiency (the local language) as a dichotomous variable (good vs. bad). Although the subjective level of oral proficiency in French may not fully reflect an objective language competency, studies found that migrants’ ability to communicate in the language of the host country was significantly and positively associated with SRH [21, 42]. We could therefore not exclude the possibility that the self-reported language competency was associated with both eligibility for regularization and SRH.

We also included variables related to social support, namely the size of the social network and the feeling of loneliness. Although the authorities did not assess the availability of social support when examining the regularization applications in the context of the “Operation Papyrus”, we could not exclude an association between social support and the eligibility for regularization. Moreover, higher social support was found to be positively associated with SRH among migrants regardless of their residence status, in Switzerland [42] and other geographical contexts [21, 43].

To measure working conditions as well as economic resources, we included respectively the number of paid working hours per week and the ability to pay an unexpected bill of CHF 1500.- (Euro 1300.-, $ 1500.-) at short notice. This last measure, intending to reflect financial resources accumulated over time, was adapted from Swiss household surveys. Because such resources may be associated with SRH [21] and reflect financial independence, an important criterion for local authorities, we deemed it appropriate to include a measure for them.

#### Control variables related to health

Chronic diseases are frequent and often cumulate among undocumented migrants [9], besides physical conditions are associated with SRH. Therefore, we included a variable related to multi-morbidity. Participants were asked about suffering from a selection of common physical chronic conditions in accordance with the Swiss Health Survey [44]. The presence of 3 or more chronic conditions defined multi-morbidity [45].

Poor mental health has also been consistently found to have a significant and negative effect on SRH. Therefore, we included a variable related to anxiety measured by the Generalized Anxiety Disorder scale (GAD-7), a validated screening questionnaire used to detect symptoms of anxiety [46]. Scores range between 0 and 27, 0 to 4 indicating no symptom of anxiety, 5 to 27 mild to severe anxiety symptoms.

Like SRH, both measures of multi-morbidity and symptoms of anxiety were self-reported. However, since they were intended to measure physical or mental health respectively and could not be assumed to share the same attributes as SRH that make the latter a comprehensive measure of health, we considered it relevant to include them as control variables.

Finally, we included a dichotomous variable related to healthcare utilization in the previous 12 months (No vs. Yes).

### Statistical analyses

Continuous variables were presented as medians and interquartile ranges and compared using Mann-Whitney’s U-test. Categorical variables were presented as absolute numbers and proportions and compared using the chi-square test. Effect sizes were measured using Cliff’s Delta or Cramer’s V, as appropriate.

We first conducted bivariate analyses between the eligibility for regularization and the other variables, including SRH. We then fitted three logistic regression models. The first model included eligibility status, sex, age, origin, recruitment site, education and experience of discrimination. We added the variables related to integration, social support, work and economic resources into a second model. Finally, we introduced the control variables related to multi-morbidity, anxiety and healthcare utilization into a third model. We set statistical significance with an alpha error of 0.05. Results of logistic regressions were presented as adjusted odds ratio (aOR) and 95% confidence interval (95% CI). All analysis were conducted using R (version 3.5.3).

## Results

### Sample characteristics

**Fig. 1:**
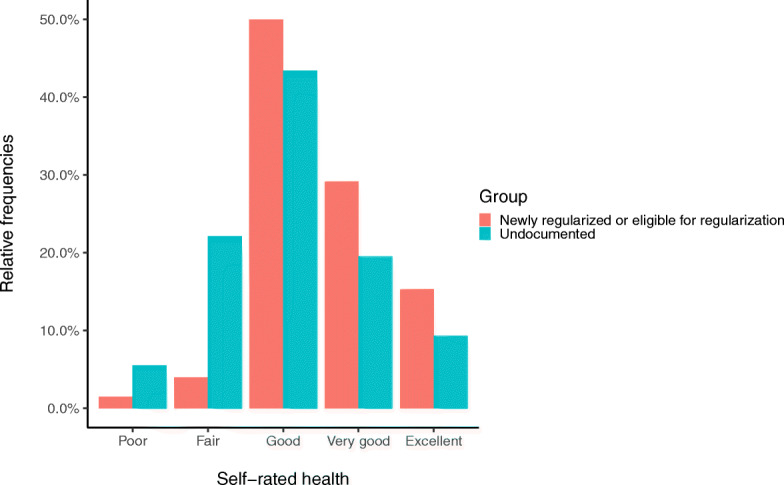
Self-rated health by eligibility status. Self-rated health is presented on its 5-point scale

We included 437 out of the 464 study participants in the analysis due to missing values in 27 who were however not significantly different in terms of demographic, socioeconomic and health status. Table 1 summarizes the demographic, socioeconomic and health-related characteristics of the participants. The sample predominantly included middle-aged women from Latin America having lived in Geneva for more than 10 years. The undocumented participants were significantly younger, had lived in Geneva for a shorter period of time and were less fluent in French as compared to the migrants in the regularized or eligible for regularization group. The newly regularized migrants and the migrants eligible for regularization could cover a CHF 1500.- unexpected bill more frequently, had larger social networks, felt less isolated and had been less exposed to racial discrimination than their undocumented counterparts. Furthermore, the regularized migrants and the migrants eligible for regularization reported overall better health (Fig. 1), were less frequently affected by symptoms of anxiety and had lower rates of multi-morbidity.

**Table 1:** Demographic, socioeconomic and health-related characteristics of the study population stratified by residence status

	Total	Undocumented	Newly regularized or eligible for regularization	p-value*	Cramer’s V / Cliff’s Delta
	*N* = 437	*N* = 235	*N* = 202		
	n(%) or median (IQR)	n(%) or median (IQR)	n(%) or median (IQR)		
Self-rated health				<.001	0.16
Very good to excellent	158 (36.2)	68 (28.9)	90 (44.6)		
Poor to good	279 (63.8)	167 (71.1)	112 (55.4)		
Sex				.358	
Female	313 (71.6)	164 (69.8)	149 (73.8)		
Male	124 (28.4)	71 (30.2)	53 (26.2)		
Age (years)	43 (15)	42 (14.5)	44 (16)	.006	−0.15**
Origin				.128	
Latin America	278 (63.6)	144 (61.3)	134 (66.3)		
Africa	31 (7.1)	21 (8.9)	10 (5.0)		
East Asia	89 (20.4)	53 (22.6)	36 (17.8)		
Eastern Europe	39 (8.9)	17 (7.2)	22 (10.9)		
Recruitment setting				<.001	0.36
In the Community	352 (80.5%)	158 (67.2)	194 (96)		
In Healthcare	85 (19.5%)	77 (32.8)	8 (4)		
Level of education				.462	
Primary or secondary	337 (77.1)	178 (75.7)	159 (78.7)		
University/higher education	100 (22.9)	57 (24.3)	43 (21.3)		
Racial discrimination				.005	0.13
No	295 (67.5)	145 (61.7)	150 (74.3)		
Yes	142 (32.5)	90 (38.3)	52 (25.7)		
Size of social network				.005	0.13
0–2	226 (51.7)	136 (57.9)	90 (44.6)		
3+	211 (48.3)	99 (42.1)	112 (55.4)		
Social isolation				<.001	0.25
Rather/very connected	313 (71.6)	144 (61.3)	169 (83.7)		
Rather/very lonely	124 (28.4)	91 (38.7)	33 (16.3)		
Duration in Geneva (years)	12 (7)	10 (7)	13 (5)	<.001	−0.45**
Language proficiency				.021	0.11
Excellent/very good	182 (41.6)	86 (36.6)	96 (47.5)		
Good/fair/poor	255 (58.4)	149 (63.4)	106 (52.5)		
Ability to face an unexpected bill				<.001	0.27
No	287 (65.7)	182 (77.4)	105 (52.0)		
Yes	150 (34.3)	53 (22.6)	97 (48.0)		
Working hours per week	32 (24)	25 (28)	37 (19)	<.001	−0.24**
Healthcare utilization in the past 12 months				.071	
No	110 (25.2)	51 (21.7)	59 (29.2)		
Yes	327 (74.8)	184 (78.3)	143 (70.8)		
Anxiety symptoms				<.001	0.19
No	283 (64.8)	132 (56.2)	151 (74.8)		
Yes	154 (35.2)	103 (43.8)	51 (25.2)		
Multi-morbidity				.034	0.10
No	358 (81.9)	184 (78.5)	174 (86.1)		
Yes	79 (18.1)	51 (21.7)	28 (13.9)		

*Comparisons between the undocumented and the newly regularized or eligible for regularization groups

**Use of Cliff’s Delta

IQR = interquartile range

### Factors associated with better SRH

Table 2 presents the results of the regression models about the association between eligibility for regularization and SRH. After adjustment for sociodemographic characteristics (model 1), the newly regularized migrants and the migrants eligible for regularization were significantly more likely than the undocumented ones to report better SRH (aOR: 1.63; 95% CI: 1.05–2.54). Regardless of their residence status, the migrants originating from East Asia (aOR 1.74; 95% CI: 1.03–2.91) or Eastern Europe (aOR 2.58; 95% CI: 1.17–5.83) were also significantly more likely to report being in very good or excellent health than the migrants from South America.

**Table 2:** Multivariate associations between better self-rated health and demographic, social economic and health factors

	model 1 aOR; 95% CI	p-value	model 2 aOR; 95%CI	p-value	model 3 aOR; 95%CI	p-value
Newly regularized or eligible for regularization (ref. Undocumented)	1.63 (1.05, 2.54)	.029	1.25 (0.77, 2.03)	.372	1.12 (0.67, 1.87)	.677
Male (ref. Female)	0.88 (0.51, 1.48)	.630	0.87 (0.49, 1.52)	.639	0.58 (0.32, 1.04)	.073
Age	1.01 (0.99, 1.03)	.525	1.00 (0.98, 1.03)	.740	1.00 (0.97, 1.03)	.938
Africa (ref. Latin America)	0.56 (0.20, 1.38)	.235	0.76 (0.25, 2.01)	.592	0.70 (0.22, 1.97)	.510
East Asia (ref. Latin America)	1.74 (1.03, 2.91)	.036	1.72 (0.98, 3.03)	.059	1.65 (0.90, 3.04)	.105
Eastern Europe (ref. Latin America)	2.58 (1.17, 5.83)	.020	2.07 (0.89, 4.90)	.093	1.60 (0.67, 3.93)	.294
Recruitment in healthcare (ref. in the community)	0.64 (0.34, 1.16)	.150	0.85 (0.43, 1.61)	.615	0.98 (0.48, 1.99)	.966
University/higher education (ref. Less than University)	0.82 (0.49, 1.36)	.456	0.82 (0.48, 1.38)	.453	0.71 (0.40, 1.24)	.233
Discrimination [Yes] (ref. No)	0.67 (0.42, 1.06)	.093	0.70 (0.43, 1.13)	.144	0.90 (0.53, 1.50)	.675
Duration in Geneva			0.99 (0.94, 1.04)	.742	1.00 (0.95, 1.06)	.889
Good/fair/poor language proficiency			0.67 (0.42, 1.07)	.095	0.69 (0.42, 1.12)	.134
Feeling of loneliness (ref. Rather/very connected)			0.42 (0.23, 0.72)	.002	0.58 (0.32, 1.04)	.074
Size of social network [3+] (ref. 0–2)			1.55 (1.00, 2.40)	.048	1.66 (1.04, 2.64)	.033
Ability to face unexpected bills [Yes] (ref. No)			1.75 (1.08, 2.82)	.022	1.42 (0.84, 2.37)	.186
Number of paid working hours			1.00 (0.99, 1.02)	.602	1.00 (0.99, 1.02)	.810
Anxiety symptoms [Yes] (ref. No)					0.25 (0.14, 0.44)	<.001
Multi-morbidity [Yes] (ref. No)					0.33 (0.14, 0.71)	.007
Healthcare utilization in the past 12 months [Yes] (ref. No)					0.58 (0.34, 0.99)	.044
Model fit indices						
Akaike Information Criterion (AIC)	558.69		540.19		498.71	
McFadden’s Pseudo-R2	.058		.111		.194	
Likelihood ratio test			X^2^ (6)=30.503	<.001	X^2^ (3)=47.479	<.001

aOR: adjusted odds ratio

95% CI: 95% confidence interval

When adjusted for variables measuring integration, social support and economic resources (model 2), eligibility for regularization and origins were no longer significantly associated with SRH. The migrants who reported being able to face an unexpected bill were more likely to report better SRH than the migrants unable to do so (aOR: 1.75; 95% CI: 1.08–2.82). Social support variables were significantly associated with SRH: less pronounced feeling of loneliness (aOR: 0.42; 95% CI: 0.23–0.72) and having a larger social network (aOR: 1.55; 95% CI: 1.00–2.40) were associated with better SRH.

In the fully adjusted model (model 3), health-related variables were significantly associated with better SRH. The migrants suffering from anxiety symptoms (aOR: 0.25; 95% CI: 0.14–0.44) or multi-morbidity (aOR: 0.33; 95% CI: 0.14–0.71) reported poorer health. Healthcare utilization in the past 12 months significantly reduced the odds of reporting very good or excellent SRH (aOR: 0.58; 95% CI: 0.34–0.99). The coefficient related to the size of the social network (aOR: 1.66; 95% CI: 1.04–2.64) remained significant. However, the ability to pay an unexpected bill as well as the feeling of loneliness lost their significant associations with SRH.

The likelihood ratio tests as well as the McFadden’s pseudo-R2 showed improvement of fit from a model to another. The McFadden’s pseudo-R2 of model 3, of 0.19, indicated a satisfactory model fit.

## Discussion

This study explored factors affecting undocumented economic migrants’ health at the onset of a pilot residence status regularization program in Geneva, Switzerland. To the best of our knowledge, it is the first study of its kind in the Western European region. It showed that while migrants undergoing regularization reported better health than those still undocumented, this difference was not associated with access to residence status after adjustment for social support and financial resources. We found that the financial resources were no longer significantly associated with SRH when adjusted for symptoms of anxiety, somatic multi-morbidity and healthcare utilization. However, the capacity to rely on a larger social network remained a significant predictor of better SRH in this population throughout the analysis.

Our results are consistent with previous findings in other geographical contexts, although some discrepancies can be highlighted [10, 14, 21, 22, 43]. In the Czech Republic, among a sample of 126 legal and 159 irregular migrants, neither residence status nor social support were associated with SRH [22]. However, socioeconomic disadvantages were associated with increased odds of poor SRH [22]. In a study conducted in Kazakhstan including 152 documented workers and 265 workers whose residence status was undocumented or undetermined, the availability of social support from family, friends, neighbours or co-workers protected against poorer SRH, unlike legal status [43]. In French Guyana, in a sample of 1027 immigrants of whom 14% were undocumented, residence status was no longer associated with SRH after adjustment for significant socioeconomic and psychosocial factors, including the perceived social support [21]. In a recent study conducted in Canada among 360 undocumented immigrants, poor SRH was not associated with residence status, but rather with psychological distress, financial strain resulting in unmet needs and lack of social support [47]. Finally, with regard to residence status regularization, DACA-eligible immigrants in the United States reported during focus groups that improved social support was one of the most beneficial consequence of DACA-regularization for their mental health and well-being [24]. Yet, quantitative studies specifically designed to address the impact of DACA regularization did not find any statistical evidence for an association between regularization and SRH [23]. Overall, our findings support the hypothesis that the self-assessment of one’s health in this group may be more related to the level of social support and economic stability than to the residence status per se.

From a public policy perspective, this study suggests that policies that address in priority the social and economic needs of undocumented populations might also promote their health. As a result, the lack of association, in the short term, between the eligibility for regularization and SRH does not mean that the residence status regularization is ineffective in the longer run. Rather, our results suggest that regularization policies may positively affect health if they are accompanied by measures fostering financial security, such as improved entitlement to social welfare or better access to legal and decent employments, and encouraging engagement in social networks [23, 24, 26]. However, further evidence based on longitudinal follow-up is needed in order to understand the social and economic mechanisms through which residence status regularization may affect health in the longer run.

Several limitations should be considered in the interpretation and generalizability of our results. As by definition undocumented migrants are not registered and random sampling was not feasible, chances are that our convenience sample may not be representative of the undocumented migrants’ population both in Geneva and elsewhere in Europe. Notably, we excluded former asylum seekers which may account for a substantial share of this group in other countries [1, 5]. In addition, we cannot exclude residual confounding. Finally, we could not assess relationships of causality between our factors of interest and the outcome as our analyses were cross-sectional.

However, considering that undocumented migrants are a hard-to-reach population, whose total size in Geneva is estimated at 10′000–15′000 persons including minors and failed asylum seekers [27], a sample size of 437 allowed for a fair precision in the measurements. Moreover, we implemented different strategies to draw a sample as diverse as possible, notably with the support of the main associations and trade unions locally involved for many years in defending undocumented migrants’ rights. As a result, 80.5% of the migrants included in this study were recruited in the community and not in healthcare settings, thus limiting the risk of bias towards poorer health in comparison with studies led in healthcare settings. In addition, the sociodemographic characteristics of our participants matched fairly well those of our population of interest – that is undocumented economic migrants - relayed in governmental as well as academic estimations across Switzerland and in Geneva [27, 48]. Finally, the consistent associations between the main predictors and the outcome support their validity.

## Conclusion

This study shows that in Geneva, the difference in SRH between undocumented migrants on the one hand, and newly regularized migrants or migrants eligible for regularization on the other hand was not associated with access to regularization but with (1) social support and (2) physical and mental health. Policies aiming at encouraging undocumented migrants’ inclusion and engagement in social networks may thus have positive health consequences for them. Future research should investigate long-term effects of residence status regularization on self-rated health.

## Supplementary Information

**Additional file 1.** Questionnaire used in the Parchemins Study for data collection (.pdf).

## Data Availability

The datasets generated and/or analysed during the current study are not publicly available due to the temporary embargo on data dissemination until 2023 required by the main funding agency of the study (Swiss National Fund for Scientific Research) but are available from the corresponding author on reasonable request.

## Abbreviations

SRH: self-rated health
DACA: Deferred Action for Childhood Arrivals Act
GAD-7: Generalized Anxiety Disorder Scale
aOR: adjusted odds ratio
95% CI: 95% confidence intervals
