# Supplementary material for: Self-rated health among undocumented and newly regularized migrants in Geneva: a cross-sectional study
Source: BMC Public Health. 2021 Jun 23;21:1198. doi: 10.1186/s12889-021-11239-0 (PMC8220781; doi:10.1186/s12889-021-11239-0)
Supplement: Supplementary file 1 — Additional file 1. Questionnaire used in the Parchemins Study for data collection (.pdf). [file 12889_2021_11239_MOESM1_ESM.pdf]

Interview

1) Ensure that the respondent has read and signed the consent sheet

2) Please complete the following information

1. Enter the ID of the interviewee

3 chiffres (c.f. liste au dos de la tablette).
En cas de perte/oubli attribuez 999.
Reportez cet identifiant sur le feuillet d'entretien (en haut à droite) et sur la feuille de consentement

2. Indicate the date of the interview

3. Is the interviewee ...

○ A man ○ A woman

Previous Next

Socio-demographic elements

We will start by discussing some points about your accommodation and your family life in Geneva ...
I remind you that at any time if a question is bothering you, you do not have to answer it and I will move on to the next one.

4. What's your birthday ? (dd/mm/yyyy)

5. In which country were you born?

If other, specify :

Previous Next

6. What is your nationality?

If other, specify :

7. Do you have a second nationality?

○ No ○ Yes

8. What is your second nationality?

If other, specify :

9. What is your marital status (from a formal point of view)?

- ○ Single
- ○ Divorced or separated
- ○ Married
- ○ Widowed, widowed

10. Currently, are you living as a couple?

- ○ No
- ○ Yes

11. Do you live in the same home as your current spouse or partner?

- ○ No
- ○ Yes

12. What is the nationality of your spouse or partner ?

[ ]

If other, specify : [ ]

Previous | Next

13. How many children do you have ? (regardless of their current residence)

Any children of the partner are not considered here.

Previous Next

As for your first child, can you tell me ...

Enter the first name / surname in the maintenance slip

14. Date of birth (mm/yyyy)

15. His country of birth

If other, specify :

16. His current country of residence

If other, specify :

17. Does he live with you in the same accomodation?

○ No ○ Yes

18. Currently, is he attending school in Geneva?

○ No ○ Yes

Previous Next

Concerning your 2nd child, can you tell me ...

Enter the first name / surname in the maintenance slip

19. Date of birth (mm/yyyy)

20. His country of birth

If other, specify :

21. His current country of residence

If other, specify :

22. Does he live with you in the same accomodation?

○ No ○ Yes

23. Currently, is he attending school in Geneva?

○ No ○ Yes

Concerning your 3rd child, can you tell me ...

Enter the first name / surname in the maintenance slip

24. Date of birth (mm/yyyy)

25. His country of birth

If other, specify :

26. His current country of residence

If other, specify :

27. Does he live with you in the same accomodation?

○ No ○ Yes

28. Currently, is he attending school in Geneva?

○ No ○ Yes

Previous Next

Concerning your 4th child, can you tell me ...

Enter the first name / surname in the maintenance slip

29. Date of birth (mm/yyyy)

30. His country of birth

If other, specify :

31. His current country of residence

If other, specify :

32. Does he live with you in the same accomodation?

○ No ○ Yes

33. Currently, is he attending school in Geneva?

○ No ○ Yes

Previous Next

Concerning your 5th child, can you tell me ...

Enter the first name / surname in the maintenance slip

34. Date of birth (mm/yyyy)

35. His country of birth

If other, specify :

36. His current country of residence

If other, specify :

37. Does he live with you in the same accomodation?

○ No ○ Yes

38. Currently, is he attending school in Geneva?

○ No ○ Yes

Previous Next

Concerning your 6th child, can you tell me ...

Enter the first name / surname in the maintenance slip

39. Date of birth (mm/yyyy)

40. His country of birth

If other, specify :

41. His current country of residence

If other, specify :

42. Does he live with you in the same accomodation?

○ No ○ Yes

43. Currently, is he attending school in Geneva?

○ No ○ Yes

← Previous Next →

Concerning your 7th child, can you tell me ...

Enter the first name / surname in the maintenance slip

44. Date of birth (mm/yyyy)

45. His country of birth

If other, specify :

46. His current country of residence

If other, specify :

47. Does he live with you in the same accomodation?

○ No ○ Yes

48. Currently, is he attending school in Geneva?

○ No ○ Yes

Concerning your 8th child, can you tell me ...

Enter the first name / surname in the maintenance slip

49. Date of birth (mm/yyyy)

50. His country of birth

If other, specify :

51. His current country of residence

If other, specify :

52. Does he live with you in the same accomodation?

○ No ○ Yes

53. Currently, is he attending school in Geneva?

○ No ○ Yes

Concerning your 9th child, can you tell me ...

Enter the first name / surname in the maintenance slip

54. Date of birth (mm/yyyy)

55. His country of birth

If other, specify :

56. His current country of residence

If other, specify :

57. Does he live with you in the same accomodation?

○ No ○ Yes

58. Currently, is he attending school in Geneva?

○ No ○ Yes

Previous Next

Level of education

We will now discuss your education background

59. At what age did you complete your education?

[ ] years

60. Which of the following levels of education corresponds to your degree?

- ○ Not completed primary school
- ○ Primary school completed
- ○ Secondary school not completed (business school, learning, college, high school, etc.)
- ○ Completed secondary school (profesional school, apprenticeship, college, high school, etc.)
- ○ University or superior education
- ○ Do not know

If university not completed -> tick " completed secondary school"

61. In which country did you graduate?

[ ]

If other, specify : [ ]

← Previous Next →

62. Have you worked in your country of origin?

- ○ No
- ○ Yes

63. What was your last job in your country of origin?

64. What was your situation in this paid activity?

- ○ Employed
- ○ Independent(e)
- ○ Employed in family business
- ○ Other
- ○ Do not know

← Previous   Next →

Housing

We will now talk about your current accomodation in Geneva

65. How many people, who you consider to be part of your family, live with you (in the same accommodation in Geneva)?

Do not count ego in the answer -> if ego lives alone, the answer is 0
Include children and spouse who may have been previously reported

66. Among these people, live with ...

	No	Yes
Your mother	○	○
Your father	○	○
Your mother in law	○	○
Your father in law	○	○

67. How many years have you been living in your current home?

[ ] years

If for less than a year -> enter 0

68. Your last move was either a choice or constrained by external factors (overpriced housing, limited time, etc.)

○ Personal choices ○ Choice constraints

69. Outside your family, with how many people do you share?

These are people who were not cited as part of the family nucleus

70. Can you tell me if your accommodation is:

- ○ In poor condition
- ○ In average condition
- ○ In good condition
- ○ New

71. How many rooms do you have (not including the kitchen, bathroom and toilet) **?**

Previous Next

72. Are you facing any of the following problems with your accommodation?

	No	Yes
Accommodation too small	○	○
Accommodation not heated well enough	○	○
Accommodation too hot (eg. under the roof during the summer)	○	○
Too much noise from neighbors or outside (road traffic, businesses, factories, etc.).	○	○
Pollution problems (related to traffic or industry, for example)	○	○

73. If 0 means 'not at all satisfied' and 10 'completely satisfied'? (Investigator), present the answer modalities A):

What is your overall level of satisfaction with your current housing?

0	1	2	3	4	5	6	7	8	9	10

Previous Next

74. With regard to your current accomodation, is it:

- ○ sublet
- ○ accommodation provided by your employer (= at home or elsewhere, but included in the employment contract)
- ○ lease at your own name or that of your spouse
- ○ [illegible]

75. What is the total rent of the dwelling? (Distinguish what ego pays and the total amount)

[ ] CHF

If ego does not know, indicate -1

76. How much do you pay per month for your rent (including charges)?

[ ] CHF

If ego does not know, indicate -1

← Previous Next →

Trajectories of migration

We will now address your migration history

77. In what year did you leave your country of origin (to <u>move</u> to another country)?

78. In what year did you arrive?

In Switzerland	
In Geneva	

79. In total, how long have you lived in Geneva?

years

Indicate the number of years

80. Have you had a residency permit (other than a visa) since your arrival in Switzerland ? (before the Papyrus regularization)

○ No ○ Yes

For example, a permit B, L (short-term authorization), F (provisionally admitted), N (asylum seeker)

Previous Next

81. For what reason(s) did you leave your country of origin (starting with the most important)
(Investigator), present the terms and conditions (B)

MAXIMUM 3 answers (4 if the respondent insists)

- Economic reasons
- Political, religious, ethnic, sexual orientation
- Family conflicts
- To ensure a better future for my loved ones
- For health reasons
- To follow or join someone
- To study
- Other

Drag and drop your answers here, and order them

If other, specify :

Previous | Next

82. Have you returned to your country of origin since settling in Switzerland?

○ No ○ Yes

83. How many times ?

[ ]

Previous | Next

84. Have you started a regularization procedure for Papyrus?

○ No ○ Yes

Si le répondant a été régularisé avant février 2017, indiquez "oui" (même si cela ne s'appelait pas encore Papyrus)

85. At what stage is the regularization procedure?

○ In preparation
○ File submitted to the authorities
○ Answer received

86. What is the answer ?

○ Negative ○ Positive

87. What is the date of your B permit ? (mm/yyyy)

88. Enquêteur(trice): ego a-t-il(elle) obtenu son permis B au cours des 3 derniers mois ? (Faire le calcul au jour exact)

Aide-mémoire:

Aujourd'hui = XX.11.2017 -> date d'obtention du permis B >= XX.08.2017 ->oui

Aujourd'hui = XX.12.2017 -> date d'obtention du permis B >= XX.09.2017 ->oui

Aujourd'hui = XX.01.2018 -> date d'obtention du permis B >= XX.10.2017 ->oui

Aujourd'hui = XX.02.2018 -> date d'obtention du permis B >= XX.11.2017 ->oui

Aujourd'hui = XX.03.2018 -> date d'obtention du permis B >= XX.12.2017 ->oui

Aujourd'hui = XX.04.2018 -> date d'obtention du permis B >= XX.01.2018 ->oui

- Oui
- Non (obtention du permis B plus ancienne)

Health

We will now discuss in more detail some points concerning your health

89. What is your size?

[ ] meters

If ego does not know and you are not in a public place, use the meter
Estimate the height of shoes (soles, heels) and subtract this value from the answer

90. What is your weight ?

[ ] kilos

If ego does not know and you are not in a public place, use the balance
Weigh ego with the shoes without the jacket

Previous | Next

I will tell you a series of health problems.

Can you tell me if they are of concern to you or if someone working in the medical field told you that you are suffering from these diseases (even if they do not bother you right now).

91.

	Disease		For how many years ?	Do you take medication?	
	No	Yes		No	Yes
Asthma					
Chronic lung disease (bronchitis_emphysema)					
Heart disease (infarction or angina, heart failure)					
High blood pressure (hypertension)					
Stroke					
Joint pain (osteoarthritis, arthritis)					
Chronic back pain and lumbar region					
Chronic pain in the neck					
Diabetes					
High cholesterol					
Osteoporosis					
Allergy (hay fever, conjunctivitis, eczema)					
Chronic liver disease					
Chronic kidney disease (do not include urinary tract infection / cystitis)					
Depression or anxiety					

92. Usually, during your leisure time, do you have a physical activity of at least 30 min that makes you sweat? (fast walking, running, cycling, etc.)

○ No ○ Yes ○ Do not know

93. On average, how many days per week?

[ ] days

The answer must be between 1 and 7

← Previous Next →

Quality of Sleep

94. During the last month, how many hours did you <u>sleep</u> on average per night?
(This number may be different from the number of hours you spent in bed)

☐ hours per night

95. During the last month, how do you evaluate the overall quality of your sleep?

Very good	Fairly good	Bad	Very bad
○	○	○	○

96. During the past month, how many times did you take medications to sleep? (prescribed by your doctor or purchased without a prescription)

Never	Less than once a week	1-2 times a week	3-4 times a week
○	○	○	○

97. During the past month, how many times did you fall asleep unintentionally during the day?

Never	Less than once a week	1-2 times a week	3-4 times a week
○	○	○	○

Health care access

98. Do you have a health insurance?

○ No ○ Yes ○ (Do not know)

99. What is the monthly premium for your health insurance? (how much do you pay per month for this insurance?)

[ ] CHF

Si ego ne sait pas, indiquez -1

Previous Next

100. What is the amount of the (annual) deductible for this insurance?

- ○ 300 CHF
- ○ 500 CHF
- ○ 1000 CHF
- ○ 1500 CHF
- ○ 2000 CHF
- ○ 2500 CHF
- ○ (Do not know)

101. Are you currently receiving subsidies for your basic health insurance premiums (LAMal)?

○ No ○ Yes ○ (Do not know)

Basic illness insurance (LAmal) for children

With regard to your children (residents in Switzerland), can you tell me if they have health insurance?

102. Child 1: Does he or she have basic health insurance (LAmal)?

○ No ○ Yes ○ Do not know

Refer to the first name of child 1 noted in the maintenance sheet

103. Child 1: What is the monthly premium for her health insurance?

[ ] CHF

Si ego ne sait pas, indiquez -1

Previous Next

104. Child 1: What is the amount of his health insurance deductible (basic LAMal) each month?

- ◯ 0 CHF
- ◯ 100 CHF
- ◯ 200 CHF
- ◯ 300 CHF
- ◯ 400 CHF
- ◯ 500 CHF
- ◯ 1000 CHF
- ◯ 1500 CHF
- ◯ 2000 CHF
- ◯ 2500 CHF
- ◯ Do not know

If the child is a minor, deductibles may be: 0, 100, 200, 300, 500 (CHF)
If the child is of age, the deductibles may be 300, 500, 1000, 1500 , 2500 (CHF)

105. Child 1: Are you currently receiving subsidies for basic health insurance premiums (LAMal)?

◯ No ◯ Yes ◯ Do not know

106. Child 2: Does he or she have basic health insurance (LAmal)?

○ No ○ Yes ○ Do not know

Refer to the first name of child 2 noted in the maintenance sheet

107. Child 2: What is the monthly premium for his health insurance?

[ ] CHF

Si ego ne sait pas, indiquez -1

Previous Next

108. Child 2: What is the amount of his / her annual health insurance deductible (basic LAMal)?

- ○ 0 CHF
- ○ 100 CHF
- ○ 200 CHF
- ○ 300 CHF
- ○ 400 CHF
- ○ 500 CHF
- ○ 1000 CHF
- ○ 1500 CHF
- ○ 2000 CHF
- ○ 2500 CHF
- ○ Do not know

If the child is a minor, deductibles may be: 0, 100, 200, 300, 500 (CHF)
If the child is of age, the deductibles may be 300, 500, 1000, 1500 , 2500 (CHF)

109. Child 2: Are you currently receiving subsidies for basic health insurance premiums (LAMal)?

○ No ○ Yes ○ Do not know

110. Child 3: Does he or she have basic health insurance (LAmal)?

○ No ○ Yes ○ Do not know

Refer to the first name of the child 3 noted in the maintenance sheet

111. Child 3: What is the monthly premium for her health insurance?

[ ] CHF

Si ego ne sait pas, indiquez -1

Previous Next

112. Child 3: What is the amount of his annual health insurance deductible (basic LAMal)?

- ○ 0 CHF
- ○ 100 CHF
- ○ 200 CHF
- ○ 300 CHF
- ○ 400 CHF
- ○ 500 CHF
- ○ 1000 CHF
- ○ 1500 CHF
- ○ 2000 CHF
- ○ 2500 CHF
- ○ Do not know

If the child is a minor, deductibles may be: 0, 100, 200, 300, 500 (CHF)
If the child is of age, the deductibles may be 300, 500, 1000, 1500 , 2500 (CHF)

113. Child 3: Do you currently receive subsidies for basic health insurance premiums (LAMal)?

○ No ○ Yes ○ Do not know

Previous Next

114. Child 4: Does he or she have basic health insurance (LAmal)?

○ No ○ Yes ○ Do not know

Refer to the first name of the child 4 noted in the maintenance sheet

115. Child 4: What is the monthly premium for her health insurance?

[ ] CHF

Si ego ne sait pas, indiquez -1

Previous Next

116. Child 4: What is the amount of his annual health insurance deductible (basic LAMal)?

- ○ 0 CHF
- ○ 100 CHF
- ○ 200 CHF
- ○ 300 CHF
- ○ 400 CHF
- ○ 500 CHF
- ○ 1000 CHF
- ○ 1500 CHF
- ○ 2000 CHF
- ○ 2500 CHF
- ○ Do not know

If the child is a minor, deductibles may be: 0, 100, 200, 300, 500 (CHF)
If the child is of age, the deductibles may be 300, 500, 1000, 1500 , 2500 (CHF)

117. Child 4: Are you currently receiving subsidies for basic health insurance premiums (LAMal)?

○ No ○ Yes ○ Do not know

118. Child 5: Does he or she have basic health insurance (LAmal)?

○ No ○ Yes ○ Do not know

Refer to the first name of the child 5 noted in the maintenance sheet

119. Child 5: What is the monthly premium for her health insurance?

[ ] CHF

Si ego ne sait pas, indiquez -1

← Previous Next →

120. Child 5: What is the amount of his annual health insurance deductible (basic LAMal)?

- ○ 0 CHF
- ○ 100 CHF
- ○ 200 CHF
- ○ 300 CHF
- ○ 400 CHF
- ○ 500 CHF
- ○ 1000 CHF
- ○ 1500 CHF
- ○ 2000 CHF
- ○ 2500 CHF
- ○ Do not know

If the child is a minor, deductibles may be: 0, 100, 200, 300, 500 (CHF)
If the child is of age, the deductibles may be 300, 500, 1000, 1500 , 2500 (CHF)

121. Child 5: Are you currently receiving subsidies for basic health insurance premiums (LAMal)?

○ No ○ Yes ○ Do not know

122. Child 6: Does he or she have basic health insurance (LAmal)?

○ No ○ Yes ○ Do not know

Refer to the first name of child 6 noted in the maintenance sheet

123. Child 6: What is the monthly premium for her health insurance?

[ ] CHF

Si ego ne sait pas, indiquez -1

Previous Next

124. Child 6: What is the amount of his annual health insurance deductible (basic LAMal)?

- ○ 0 CHF
- ○ 100 CHF
- ○ 200 CHF
- ○ 300 CHF
- ○ 400 CHF
- ○ 500 CHF
- ○ 1000 CHF
- ○ 1500 CHF
- ○ 2000 CHF
- ○ 2500 CHF
- ○ Do not know

If the child is a minor, deductibles may be: 0, 100, 200, 300, 500 (CHF)
If the child is of age, the deductibles may be 300, 500, 1000, 1500 , 2500 (CHF)

125. Child 6: Are you currently receiving subsidies for basic health insurance premiums (LAMal)?

○ No ○ Yes ○ Do not know

126. Child 7: Does he or she have basic health insurance (LAmal)?

○ No ○ Yes ○ Do not know

Refer to the first name of the child 7 noted in the maintenance sheet

127. Child 7: What is the monthly premium for her health insurance?

[ ] CHF

Si ego ne sait pas, indiquez -1

← Previous Next →

128. Child 7: What is the amount of his annual health insurance deductible (basic LAMal)?

- ○ 0 CHF
- ○ 100 CHF
- ○ 200 CHF
- ○ 300 CHF
- ○ 400 CHF
- ○ 500 CHF
- ○ 1000 CHF
- ○ 1500 CHF
- ○ 2000 CHF
- ○ 2500 CHF
- ○ Do not know

If the child is a minor, deductibles may be: 0, 100, 200, 300, 500 (CHF)
If the child is of age, the deductibles may be 300, 500, 1000, 1500 , 2500 (CHF)

129. Child 7: Are you currently receiving subsidies for basic health insurance premiums (LAMal)?

○ No ○ Yes ○ Do not know

130. Child 8: Does he or she have basic health insurance (LAmal)?

○ No ○ Yes ○ Do not know

Refer to the first name of child 8 noted in the maintenance sheet

131. Child 8: What is the monthly premium for her health insurance?

[ ] CHF

Si ego ne sait pas, indiquez -1

← Previous Next →

132. Child 8: What is the amount of his annual health insurance deductible (basic LAMal)?

- ○ 0 CHF
- ○ 100 CHF
- ○ 200 CHF
- ○ 300 CHF
- ○ 400 CHF
- ○ 500 CHF
- ○ 1000 CHF
- ○ 1500 CHF
- ○ 2000 CHF
- ○ 2500 CHF
- ○ Do not know

If the child is a minor, deductibles may be: 0, 100, 200, 300, 500 (CHF)
If the child is of age, the deductibles may be 300, 500, 1000, 1500 , 2500 (CHF)

133. Child 8: Are you currently receiving subsidies for basic health insurance premiums (LAMal)?

○ No ○ Yes ○ Do not know

134. Child 9: Does he or she have basic health insurance (LAmal)?

○ No ○ Yes ○ Do not know

Refer to the child's first name 9 in the maintenance sheet

135. Child 9: What is the monthly premium for her health insurance?

[ ] CHF

Si ego ne sait pas, indiquez -1

136. Child 9: What is the amount of his annual health insurance deductible (basic LAMal)?

- ○ 0 CHF
- ○ 100 CHF
- ○ 200 CHF
- ○ 300 CHF
- ○ 400 CHF
- ○ 500 CHF
- ○ 1000 CHF
- ○ 1500 CHF
- ○ 2000 CHF
- ○ 2500 CHF
- ○ Do not know

If the child is a minor, deductibles may be: 0, 100, 200, 300, 500 (CHF)
If the child is of age, the deductibles may be 300, 500, 1000, 1500 , 2500 (CHF)

137. Child 9: Are you currently receiving subsidies for basic health insurance premiums (LAMal)?

○ No ○ Yes ○ Do not know

Previous Next

Basic illness insurance (LAmal) of other members of the household

138. Does your spouse have basic health insurance (LAmal)?

○ No ○ Yes ○ (Do not know)

139. Spouse: What is the amount of his / her monthly health insurance premium?

[ ] CHF per month

If ego does not know, indicate -1

← Previous Next →

140. Spouse: What is the annual amount of the insurance deductible?

- 300 CHF
- 500 CHF
- 1000 CHF
- 1500 CHF
- 2000 CHF
- 2500 CHF
- (Do not know)

141. Spouse: Is he currently receiving subsidies for his basic health insurance premiums (LAMal)?

- No
- Yes
- (Do not know)

142. Does your mother have basic health insurance (LAmal)?

○ No ○ Yes ○ (Do not know)

143. Mother: What is the amount of her monthly health insurance premium?

[ ] CHF per month

If ego does not know, indicate -1

144. Mother: Is she currently receiving subsidies for her basic health insurance premiums (LAMal)?

○ No ○ Yes ○ (Do not know)

145. Does your father have basic health insurance (LAmal)?

○ No ○ Yes ○ (Do not know)

146. Father: What is the amount of his monthly health insurance premium?

[ ] CHF per month

If ego does not know, indicate -1

147. Father: Is she currently receiving subsidies for her basic health insurance premiums (LAMal)?

○ No ○ Yes ○ (Do not know)

148. Does your mother-in-law have basic health insurance (LAmal)?

○ No ○ Yes ○ (Do not know)

149. Mother-in-law: What is the amount of her monthly health insurance premium?

[ ] CHF per month

If ego does not know, indicate -1

150. Mother-in-law: Is she currently receiving subsidies for her basic health insurance premiums (LAMal)?

○ No ○ Yes ○ (Do not know)

Previous Next

151. Does your father in law have basic health insurance (LAmal)?

○ No ○ Yes ○ (Do not know)

152. father in law: What is the amount of his monthly health insurance premium?

[ ] CHF per month

If ego does not know, indicate -1

153. father in law: Is he currently receiving subsidies for his basic health insurance premiums (LAMal)?

○ No ○ Yes ○ (Do not know)

Access to care, continued

154. Do you have a family doctor?

(That is, a doctor to whom you can go for most of your health problems)

○ No ○ Yes

155. Is it a doctor ...

○ private, outside the hospital ○ at CAMSCO (Hugo) or HUG ○ (Do not know)

156. During the past 12 months, how often have you visited a doctor (general practitioner, specialist, psychiatrist or gynecologist), not including a dentist**?**

Previous Next

157. In the past 12 months, how often have you consulted in a emergency service?

158. In the past 12 months, were you hospitalized for more than 24 hours?

○ No ○ Yes ○ (Do not know)

159. How many times ?

Previous Next

160. During the past 6 months, did you have an accident that prevented you from working?

○ No ○ Yes

161. In what context ?

○ At work
○ At home
○ During your leisure time, sport
○ Outside your home
○ Other

162. In the past 12 months, have you renounced to seek health care or to buy equipment because of the price to pay?

- ○ No
- ○ Yes
- ○ (Do not know)

163. What kind of care have you given up?

- ☐ A surgical procedure
- ☐ Dental care
- ☐ Physical therapy (physio, osteopathy)
- ☐ Medical care with a general practitioner
- ☐ Radiology (x-ray, scanner, ultrsound)
- ☐ Buyng glasses, hearing aid device, crutches, etc.
- ☐ Medical care with a gynecologist
- ☐ Buying medicine / prescription drugs
- ☐ Home care with nurses
- ☐ Medical care with a specialist (cardiologist, pneumologist, etc.)
- ☐ Rehabilitation at hospital
- ☐ Other

If other, specify : ____________________

Make sure that the motivation to give up this care has been financial.
MULTIPLE ANSWERS POSSIBLE

Health more ...

164. In the past <u>two weeks</u> , how often have you been disturbed by the following problems? **(Investigator, present the answer modalities C)**

	Never	Rarely	Often	Almost every day
Little interest or pleasure in doing things	○	○	○	○
You feel sad, depressed or desperate	○	○	○	○
Having difficulty falling asleep or staying asleep, or sleeping too much	○	○	○	○
Feeling tired or having little energy	○	○	○	○
Have little appetite or overeating	○	○	○	○
Having low self-esteem	○	○	○	○
Having difficulty concentrating	○	○	○	○
Your loved ones tell you that you are particularly agitated or slowed down	○	○	○	○
You thought you'd hurt yourself or you'd be better off dead	○	○	○	○

165. In the past <u>two</u> weeks, how often have you been disturbed by the following problems? **(Investigator, present the answer modalities C)**

	Never	Rarely	Often	Almost every day
Feelings of nervousness, anxiety or tension	○	○	○	○
Being unable to control your concerns	○	○	○	○
Worrying about everything	○	○	○	○
Difficulty to relax	○	○	○	○
Restlessness such that it is difficult to remain quiet	○	○	○	○
Becoming easily angry or irritable	○	○	○	○
Be afraid that something terrible may happen	○	○	○	○

← Previous Next →

166. Overall, do you think your health is ...

Excellent	Very good	Good	Fair	Poor
○	○	○	○	○

167. Compared to the Swiss population, do you think your health is ...
(Investigator, present the answer modalities D)

Much better	Better	Identical	Worse	Really worse	Do not know
○	○	○	○	○	○

168. Compared to other undocumented migrants in Geneva, do you think your health is ...
(Investigator, present the answer modalities D)

Much better	Better	Identical	Worse	Really worse	Do not know
○	○	○	○	○	○

169. Compared to the population in your country of origin, do you think your health is...
Investigator, present the answer modalities D)

Much better	Better	Identical	Worse	Really worse	Do not know
○	○	○	○	○	○

← Previous Next →

Economy and occupational status

170. On average, how many hours do you usually work per week in your paid work?

[ ]

If ego does not know, indicate temporal reference of the last 3 months

171. Why are you currently unemployed ?

- ☐ Recent job loss
- ☐ Health problem, accident
- ☐ Other

If other, specifiy [ ]

172. How many employers do you have?

[ ]

173. What is your approximate total monthly salary?

[ ] CHF

This is the total monthly income received by cumulating all the jobs currently occupied

174. Are you registered at Chèque Service ?

○ No ○ Yes ○ Do not know

Previous Next

So, a little more detailed, can you provide me with the following information about each of your jobs ...

Interviewer, use the "employeurs" section of the maintenance sheet as an aide-mémoire if necessary

175. Employer 1: What is the type of this job?

- ○ Domestic work (household, personal assistance, gardening, etc.)
- ○ Building
- ○ Hotels / Catering / cooking in a restaurant
- ○ Industry
- ○ Other

If other, specify : ______

176. Employer 1: How many hours per week do you work for this job?

______

177. Employer 1: What wage do you earn with this job?

The question is relevant only if employeurs_nb >= 1.

Hourly wage (per hour)		CHF
Monthly pay		CHF

Previous Next

178. Employer 1: Is this job declared by your employer (are they paid social contributions) **?**

○ No ○ Yes ○ (Do not know)

179. Employer 1: How long have you been employed? (mm/yyyy)

180. Employer 1: Does this "new" job correspond to a change of type of activity?

○ No ○ Yes

Est-ce que cet emploi (occupé depuis env. 6 mois) a remplacé un emploi occupé dans un secteur d'activité différent ? (secteur domestique -> hôtellerie,restauration par ex)

181. Employer 1: What was the type of the previous job?

○ Domestic work (household, personal assistance, gardening, etc.)
○ Building
○ Hotels / Catering / cooking in a restaurant
○ Industry
○ Other

If other, specify :

Previous Next

182. Employer 2: What is the sector of this job?

- ○ Domestic work (household, personal assistance, gardening, etc.)
- ○ Building
- ○ Hotels / Catering / cooking in a restaurant
- ○ Industry
- ○ Other

If other, specify : ____________________

183. Employer 2: How many hours per week do you work for this job?

____

184. Employer 2: What wage do you earn with this job?

The question is relevant only if employeurs_nb >= 2.

Hourly wage (per hour)	______	CHF
Monthly pay	______	CHF

← Previous   Next →

185. Employer 2: Is this job declared by your employer (are the social contributions paid) **?**

○ No ○ Yes ○ (Do not know)

186. Employer 2: How long have you been employed? (mm/yyyy)

187. Employer 2: Does this "new" job correspond to a change of type of activity?

○ No ○ Yes

Est-ce que cet emploi (occupé depuis env. 6 mois) a remplacé un emploi occupé dans un secteur d'activité différent ? (secteur domestique -> hôtellerie,restauration par ex)

188. Employer 2: What was the type of the previous job?

○ Domestic work (household, personal assistance, gardening, etc.)
○ Building
○ Hotels / Catering / cooking in a restaurant
○ Industry
○ Other

If other, specify :

Previous Next

189. Employer 3: What is the activity of this job?

- ○ Domestic work (household, personal assistance, gardening, etc.)
- ○ Building
- ○ Hotels / Catering / cooking in a restaurant
- ○ Industry
- ○ Other

If other, specify : [ ]

190. Employer 3: How many hours per week do you work for this job?

[ ]

191. Employer 3: What wage do you earn with this job?

The question is relevant only if employeurs_nb >= 3.

Hourly wage (per hour)	[ ]	CHF
Monthly pay	[ ]	CHF

Previous | Next

192. Employer 3: Is this job declared by your employer (are the social contributions paid) **?**

○ No ○ Yes ○ (Do not know)

193. Employer 3: How long have you been employed? (mm/yyyy)

194. Employer 3: Does this "new" job correspond to a change of type of activity?

○ No ○ Yes

Est-ce que cet emploi (occupé depuis env. 6 mois) a remplacé un emploi occupé dans un secteur d'activité différent ? (secteur domestique -> hôtellerie,restauration par ex)

195. Employer 3: What was the type of the previous job?

○ Domestic work (household, personal assistance, gardening, etc.)
○ Building
○ Hotels / Catering / cooking in a restaurant
○ Industry
○ Other

If other, specify :

Previous Next

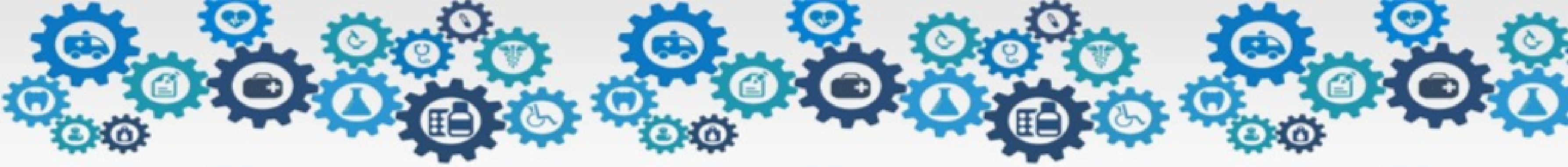

196. Employer 4: What is the sector of this job?

- ○ Domestic work (household, personal assistance, gardening, etc.)
- ○ Building
- ○ Hotels / Catering / cooking in a restaurant
- ○ Industry
- ○ Other

If other, specify : ______

197. Employer 4: How many hours per week do you work for this job?

______

198. Employer 4: What wage do you earn with this job?

The question is relevant only if employeurs_nb >= 4.

Hourly wage (per hour)	______	CHF
Monthly pay	______	CHF

← Previous | Next →

199. Employer 4: Is this job declared by your employer (are they paid social contributions) **?**

○ No ○ Yes ○ (Do not know)

200. Employer 4: How long have you been employed? (mm / yyyy)

201. Employer 4: Does this "new" job correspond to a change of type of activity?

○ No ○ Yes

Est-ce que cet emploi (occupé depuis env. 6 mois) a remplacé un emploi occupé dans un secteur d'activité différent ? (secteur domestique -> hôtellerie,restauration par ex)

202. Employer 4: What was the type of the previous job?

○ Domestic work (household, personal assistance, gardening, etc.)
○ Building
○ Hotels / Catering / cooking in a restaurant
○ Industry
○ Other

If other, specify :

Previous Next

203. Employer 5: What is the sector of this job?

- ○ Domestic work (household, personal assistance, gardening, etc.)
- ○ Building
- ○ Hotels / Catering / cooking in a restaurant
- ○ Industry
- ○ Other

If other, specify : __________

204. Employer 5: How many hours per week do you work for this job?

____

205. Employer 5: What wage do you earn with this job?

The question is relevant only if employeurs_nb >= 5.

Hourly wage (per hour)		CHF
Monthly pay		CHF

Previous | Next

206. Employer 5: Is this job declared by your employer (are the social contributions paid) **?**

○ No ○ Yes ○ (Do not know)

207. Employer 5: How long have you been employed? (mm/yyyy)

208. Employer 5: Does this "new" job correspond to a change of type of activity?

○ No ○ Yes

Est-ce que cet emploi (occupé depuis env. 6 mois) a remplacé un emploi occupé dans un secteur d'activité différent ? (secteur domestique -> hôtellerie,restauration par ex)

209. Employer 5: What was the type of the previous job?

○ Domestic work (household, personal assistance, gardening, etc.)
○ Building
○ Hotels / Catering / cooking in a restaurant
○ Industry
○ Other

If other, specify :

Previous Next

210. Employer 6: What is the activity of this job?

- ◯ Domestic work (household, personal assistance, gardening, etc.)
- ◯ Building
- ◯ Hotels / Catering / cooking in a restaurant
- ◯ Industry
- ◯ Other

If other, specify : ____________________

211. Employer 6: How many hours per week do you work for this job?

____

212. Employer 6: What wage do you earn with this job?

The question is relevant only if employeurs_nb >= 6.

Hourly wage (per hour)		CHF
Monthly pay		CHF

← Previous | Next →

213. Employer 6: Is this job declared by your employer (are they paid social contributions) **?**

○ No ○ Yes ○ (Do not know)

214. Employer 6: How long have you been employed? (mm/yyyy)

215. Employer 6: Does this "new" job correspond to a change of type of activity?

○ No ○ Yes

Est-ce que cet emploi (occupé depuis env. 6 mois) a remplacé un emploi occupé dans un secteur d'activité différent ? (secteur domestique -> hôtellerie,restauration par ex)

216. Employer 6: What was the type of the previous job?

○ Domestic work (household, personal assistance, gardening, etc.)
○ Building
○ Hotels / Catering / cooking in a restaurant
○ Industry
○ Other

If other, specify :

Previous Next

217. Employer 7: What is the activity of this job?

- ○ Domestic work (household, personal assistance, gardening, etc.)
- ○ Building
- ○ Hotels / Catering / cooking in a restaurant
- ○ Industry
- ○ Other

If other, specify : ______

218. Employer 7: How many hours per week do you work for this job?

______

219. Employer 7: What wage do you earn with this job?

The question is relevant only if employeurs_nb >= 7.

Hourly wage (per hour)	______ CHF
Monthly pay	______ CHF

Previous   Next

220. Employer 7: Is this job declared by your employer (are they paid social contributions) **?**

○ No ○ Yes ○ (Do not know)

221. Employer 7: How long have you been employed? (mm/yyyy)

222. Employer 7: Does this "new" job correspond to a change of type of activity?

○ No ○ Yes

Est-ce que cet emploi (occupé depuis env. 6 mois) a remplacé un emploi occupé dans un secteur d'activité différent ? (secteur domestique -> hôtellerie,restauration par ex)

223. Employer 7: What was the type of the previous job?

○ Domestic work (household, personal assistance, gardening, etc.)
○ Building
○ Hotels / Catering / cooking in a restaurant
○ Industry
○ Other

If other, specify :

Previous Next

224. Employer 8: What is the activity sector of this job?

- ○ Domestic work (household, personal assistance, gardening, etc.)
- ○ Building
- ○ Hotels / Catering / cooking in a restaurant
- ○ Industry
- ○ Other

If other, specify : ______

225. Employer 8: How many hours per week do you work for this job?

______

226. Employer 8: What wage do you earn with this job?

The question is relevant only if employeurs_nb >= 8.

Hourly wage (per hour)	______	CHF
Monthly pay	______	CHF

← Previous Next →

227. Employer 8: Is this job declared by your employer (are the social contributions paid) **?**

○ No ○ Yes ○ (Do not know)

228. Employer 8: How long have you been employed? (mm/yyyy)

229. Employer 8: Does this "new" job correspond to a change of type of activity?

○ No ○ Yes

Est-ce que cet emploi (occupé depuis env. 6 mois) a remplacé un emploi occupé dans un secteur d'activité différent ? (secteur domestique -> hôtellerie,restauration par ex)

230. Employer 8: What was the type of the previous job?

○ Domestic work (household, personal assistance, gardening, etc.)
○ Building
○ Hotels / Catering / cooking in a restaurant
○ Industry
○ Other

If other, specify :

Previous Next

231. Employer 9: What is the activity of this job?

- ○ Domestic work (household, personal assistance, gardening, etc.)
- ○ Building
- ○ Hotels / Catering / cooking in a restaurant
- ○ Industry
- ○ Other

If other, specify : ____________

232. Employer 9: How many hours per week do you work for this job?

____

233. Employer 9: What wage do you earn with this job?

The question is relevant only if employeurs_nb >= 9.

Hourly wage (per hour)	____	CHF
Monthly pay	____	CHF

← Previous | Next →

234. Employer 9: Is this job declared by your employer (are they paid social contributions) **?**

○ No ○ Yes ○ (Do not know)

235. Employer 9: How long have you been employed? (mm/yyyy)

236. Employer 9: Does this "new" job correspond to a change of type of activity?

○ No ○ Yes

Est-ce que cet emploi (occupé depuis env. 6 mois) a remplacé un emploi occupé dans un secteur d'activité différent ? (secteur domestique -> hôtellerie,restauration par ex)

237. Employer 9: What was the type of the previous job?

○ Domestic work (household, personal assistance, gardening, etc.)
○ Building
○ Hotels / Catering / cooking in a restaurant
○ Industry
○ Other

If other, specify :

Previous Next

238. Employer 10: What is the sector of this job?

- ○ Domestic work (household, personal assistance, gardening, etc.)
- ○ Building
- ○ Hotels / Catering / cooking in a restaurant
- ○ Industry
- ○ Other

If other, specify :

239. Employer 10: How many hours per week do you work for this job?

240. Employer 10: What wage do you earn with this job?

The question is relevant only if employeurs_nb >= 10.

Hourly wage (per hour)		CHF
Monthly pay		CHF

Previous Next

241. Employer 10: Is this job declared by your employer (are the social contributions paid) **?**

- ○ No
- ○ Yes
- ○ (Do not know)

242. Employer 10: How long have you been employed? (mm/yyyy))

243. Employer 10: Does this "new" job correspond to a change of type of activity?

- ○ No
- ○ Yes

Est-ce que cet emploi (occupé depuis env. 6 mois) a remplacé un emploi occupé dans un secteur d'activité différent ? (secteur domestique -> hôtellerie,restauration par ex)

244. Employer 10: What was the type of the previous job?

- ○ Domestic work (household, personal assistance, gardening, etc.)
- ○ Building
- ○ Hotels / Catering / cooking in a restaurant
- ○ Industry
- ○ Other

If other, specify :

Previous | Next

245. Employer 11: What is the sector of this job?

- ○ Domestic work (household, personal assistance, gardening, etc.)
- ○ Building
- ○ Hotels / Catering / cooking in a restaurant
- ○ Industry
- ○ Other

If other, specify : __________

246. Employer 11: How many hours per week do you work for this job?

____

247. Employer 11: What wage do you earn with this job?

The question is relevant only if employeurs_nb >= 11.

Hourly wage (per hour)		CHF
Monthly pay		CHF

Previous | Next

248. Employer 11: Is this job declared by your employer (are the social contributions paid) **?**

○ No ○ Yes ○ (Do not know)

249. Employer 11: How long have you been employed? (mm/yyyy)

250. Employer 11: Does this "new" job correspond to a change of type of activity?

○ No ○ Yes

Est-ce que cet emploi (occupé depuis env. 6 mois) a remplacé un emploi occupé dans un secteur d'activité différent ? (secteur domestique -> hôtellerie,restauration par ex)

251. Employer 11: What was the type of the previous job?

○ Domestic work (household, personal assistance, gardening, etc.)
○ Building
○ Hotels / Catering / cooking in a restaurant
○ Industry
○ Other

If other, specify :

Previous Next

252. Employer 12: What is the activity of this job?

- ○ Domestic work (household, personal assistance, gardening, etc.)
- ○ Building
- ○ Hotels / Catering / cooking in a restaurant
- ○ Industry
- ○ Other

If other, specify :

253. Employer 12: How many hours per week do you work for this job?

254. Employer 12: What wage do you earn with this job?

The question is relevant only if employeurs_nb >= 12.

Hourly wage (per hour)		CHF
Monthly pay		CHF

Previous Next

255. Employer 12: Is this job declared by your employer (are social security contributions paid) **?**

- ○ No
- ○ Yes
- ○ (Do not know)

256. Employer 12: How long have you been employed? (mm/yyyy)

[ ]

257. Employer 12: Does this "new" job correspond to a change of type of activity?

- ○ No
- ○ Yes

Est-ce que cet emploi (occupé depuis env. 6 mois) a remplacé un emploi occupé dans un secteur d'activité différent ? (secteur domestique -> hôtellerie,restauration par ex)

258. Employer 12: What was the type of the previous job?

- ○ Domestic work (household, personal assistance, gardening, etc.)
- ○ Building
- ○ Hotels / Catering / cooking in a restaurant
- ○ Industry
- ○ Other

If other, specify : [ ]

Previous | Next

259. Is your spouse currently working?

○ No ○ Yes

260. In which sector does your spouse work?

○ Domestic work (household, personal assistance, gardening, etc.)
○ Building
○ Hotels / Catering / cooking in a restaurant
○ Industry
○ Other

If other, specify : __________

If several jobs indicate information for the main activity

261. What is the approximate monthly salary of your spouse?

________ CHF

262. Does your spouse have a working permit?

○ No ○ Yes ○ (Do not know)

263. Among the other members of the household, do some contribute to household income through <u>paid work in Geneva</u> ?

○ No ○ Yes

Do not consider the spouse here

264. What is, approximately, the sum of the monthly wages (of each member of the family nucleus)?

[ ] CHF

Excluindo o cônjuge e ego aqui

265. The total monthly salary of the family is therefore approximately ...

[ ] CHF

Make a balance sheet by summing the income of ego, his spouse and other family members

Previous Next

266. Are you currently receiving financial assistance? (eg. family allowances, assistance from the Hospice General, etc.)

○ No ○ Yes ○ (Do not know)

These are formal aids and not the support of relatives

267. Can you tell me the monthly (total) amount of this financial aid?

[ ] CHF

If it is easier for ego to indicate an annual amount, divide that amount by 12.

268. From whom (which institutions) do you receive these financial assistance?

[ ]

269. If all of a sudden you had to pay an invoice of 1500.-, would that be possible?

○ No ○ Yes

270. Do you send money to your family in the country of origin?

○ No ○ Yes ○ You do not have a family in the country of origin

271. On average, how much do you send per month?

[ ] CHF

If it is easier for ego to indicate an annual amount, divide that amount by 12.

272. Do you have any financial debts currently?

○ No ○ Yes

273. How much (approximatively) ?

[ ] CHF

Si ego ne sait pas -> indiquez -1

274. If 0 means 'not at all satisfied' and 10 'completely satisfied'? **(Investigator, present the answer modalities A):**

What is your overall level of satisfaction with your financial situation?

0	1	2	3	4	5	6	7	8	9	10

Previous Next

275. In recent times, have you taken steps that have failed to find one or more new jobs?

○ No ○ Yes

If ego does not know, indicate temporal reference of the last 3 months

276. For what reasons have you taken these steps?

☐ Earn more money
☐ Increase your working time
☐ Decrease your working time
☐ Reduce your number of employers
☐ Change your sector of activity
☐ Autre

Si 'Autre' précisez :

Many possible responses

277. In which area would you like to find a new job?

○ Domestic work (household, personal assistance, gardening, etc.)
○ Building
○ Hotels / Catering / cooking in a restaurant
○ Industry
○ Other

If other, specify :

Previous Next

278. Did you lose a job because of your regularization ?

- ○ No
- ○ Yes

279. If you were to lose your current position, would you find it easy or difficult to find a new comparable position?

Very easily	Fairly easily	Rather difficult	Very difficult

280. How many days per month do you work 10 hours or more?

[ ] days per month

281. Usually, how many nights per month do you work ? (for at least 2 hours between 11 o'clock in the evening and 6 o'clock in the morning)

[ ] nights per month

282. In the past 12 months, did you ever work while you were sick?

- ○ I was sick and I continued to work
- ○ I was sick and I stopped working
- ○ I was not sick

283. For how many days did it happen?

[ ] days per year

Previous | Next

284. Please indicate each time, using the following scale (Investigator), present the answer modalities (C):

How often your work involves

	Never	Rarely	Often	Almost all the time
Painful or tiring positions	○	○	○	○
Lifting or moving heavy loads	○	○	○	○
To remain standing	○	○	○	○
Repetitive movements of the hand or arm	○	○	○	○

285. Please indicate each time, using the following scale (Investigator, present the answer modalities C):

How often you are exposed to your work at:

	Never	Rarely	Often	Almost all the time
Vibrations caused by hand tools, machines, etc.	○	○	○	○
Noises so strong that you need to raise your voice to talk to people	○	○	○	○
High temperatures that make you sweat even if you do not work	○	○	○	○
Low temperatures either indoors or outdoors	○	○	○	○
Harmful or toxic products (or substances): dust, industrial smoke, microbes, chemicals, etc.	○	○	○	○
Passive smoking: Inhalation of cigarette smoke from nearby smokers	○	○	○	○

286. Does your work affect your health? (indicate the best answer)

- ☐ No
- ☐ Yes, mainly positive
- ☐ Yes, mostly negative
- ☐ (Do not know)

Previous Next

287. In relation to your work, how often do the following statements apply to your personal situation?
If 0 means 'never' and 6 'almost every day'.

	0 Never	1	2	3	4	5	6 Almost every day
You feel emotionally exhausted by your work	○	○	○	○	○	○	○
You feel very annoyed at the end of your working day	○	○	○	○	○	○	○
You feel tired when you get up in the morning and you have to face another day of work	○	○	○	○	○	○	○
Working with people throughout the day requires a lot of effort	○	○	○	○	○	○	○
You feel you may breakdown because of your work	○	○	○	○	○	○	○
You feel frustrated by your work	○	○	○	○	○	○	○
You feel that you are working 'too hard' in your work	○	○	○	○	○	○	○
Working in direct contact with people will stress you too	○	○	○	○	○	○	○
Your work exhausts you	○	○	○	○	○	○	○

288. Can you tell me on a scale from 0 'not at all satisfied' to 10 'completely satisfied' **(Investigator, present the answer modalities A) :**

Your satisfaction with each of the following?

	0	1	2	3	4	5	6	7	8	9	10
Your working conditions	○	○	○	○	○	○	○	○	○	○	○
The income you earn from your work	○	○	○	○	○	○	○	○	○	○	○
The atmosphere with co-workers	○	○	○	○	○	○	○	○	○	○	○
The interest of tasks	○	○	○	○	○	○	○	○	○	○	○
Your amount of work	○	○	○	○	○	○	○	○	○	○	○

289. In the past 12 months, have you been the subject of ...

Tick a maximum of 4 boxes.

	No	Yes, at work or in the labor market context	Yes, on the street or in a public place	Yes, in the hospital or at your doctor	Yes, in another place
discrimination related to your age	☐	☐	☐	☐	☐
discrimination related to your gender	☐	☐	☐	☐	☐
discrimination related to your nationality, ethnic origin or color	☐	☐	☐	☐	☐
discrimination linked to disability	☐	☐	☐	☐	☐
verbal abuse	☐	☐	☐	☐	☐
Threats and Humiliating Behaviors	☐	☐	☐	☐	☐
physical violence	☐	☐	☐	☐	☐
intimidation, harassment, mobbing	☐	☐	☐	☐	☐
sexual harassment	☐	☐	☐	☐	☐

290. If 0 is "not at all satisfied" and 10 "completely satisfied" (Investigator, present the answer modalities A):

Are you satisfied with your personal, family and social relationships ?

0	1	2	3	4	5	6	7	8	9	10

291. To what extent are you satisfied with living together in your household, if 0 is "not at all satisfied" and 10 "completely satisfied"? (Investigator, present the answer modalities A)

0	1	2	3	4	5	6	7	8	9	10

292. How satisfied are you with living alone in your household, if 0 is "not at all satisfied" and 10 "completely satisfied"? (Investigator, present the answer modalities A)

0	1	2	3	4	5	6	7	8	9	10

293. If necessary, can you rely on someone to help you in everyday life, give you a hand? (eg. helping you to go somewhere, for some work at home, to keep your children ...)

Whether it is a member of the household, family, friend, colleague, neighbor, etc.

- ○ No
- ○ Yes

294. How many people are close enough for you to rely on them for serious personal problems?

- ○ 0
- ○ 1-2
- ○ 3-4
- ○ 5-6
- ○ 7 or more

295. Is there anybody among your loved ones who can really talk to you at any time about very personal problems?

○ No ○ Yes, one person ○ Yes, several people

296. In general, you would say that you feel ...

Very lonely	Rather lonely	Rather connected to other people	Very connected to other people

297. How often do you participate in the activities of a society, a sports club, a political party, a cultural association, a religious community or other groups?

Almost every day	About once a week	About 1 to 3 times a month	A few times a year	More rarely	Never
○	○	○	○	○	○

298. In your opinion, what is your level of oral proficiency in French?

Very good	Good	Sufficient	Bad	Very bad	(Do not know)
○	○	○	○	○	○

299. In your opinion, what is your level of mastery of written French?

Very good	Good	Sufficient	Bad	Very bad	(Do not know)
○	○	○	○	○	○

Satisfaction with quality of life

Regarding your current life ...

300. In general, if 0 means 'not at all satisfied' and 10 'completely satisfied'? **(Investigator, present the answer modalities A) :**

How satisfied are you with your life?

0	1	2	3	4	5	6	7	8	9	10

Previous Next

301. In relation to the Swiss population, do you think your quality of life is ...
(Investigator, present the answer modalities D)

Much better	Better	Identical	Worse	Really worse	(Do not know)
○	○	○	○	○	○

302. Compared to other undocumented migrants in Geneva, do you think your quality of life is ...
(Investigator, present the answer modalities D)

Much better	Better	Identical	Worse	Really worse	(Do not know)
○	○	○	○	○	○

303. Compared to the population in your country of origin, do you think your quality of life is ...
(Investigator, present the answer modalities D)

Much better	Better	Identical	Worse	Really worse	(Do not know)
○	○	○	○	○	○

← Previous Next →

End of interview

The interview is now over. We thank you for your participation which is very valuable ...

304. Enquêteur(trice): A quel groupe de l'étude le répondant correspond-il ?

- ○ Contrôle (aucun dossier constitué / critères incomplets / dossier refusé)
- ○ Indéterminé (dossier en constitution auprès d'une association / dossier transmis aux autorités, en attente de réponse)
- ○ Papyrus (obtention d'un permis B via l'opération Papyrus au cours des 3 derniers mois)
- ○ Mandat (obtention d'un permis B AVANT la période des 3 derniers mois)

INVESTIGATOR, please click on "save" and fill in the "reprise de contact" section of the maintenance sheet.

← Previous ✔ Save
